# Infantile spasms (West syndrome): update and resources for pediatricians and providers to share with parents

**DOI:** 10.1186/1471-2431-12-108

**Published:** 2012-07-25

**Authors:** James W Wheless, Patricia A Gibson, Kari Luther Rosbeck, Maria Hardin, Christine O’Dell, Vicky Whittemore, John M Pellock

**Affiliations:** 1 Professor and Chief of Pediatric Neurology, LeBonheur Chair in Pediatric Neurology, University of Tennessee Health Science Center, Memphis, TN, USA; 2 Director, LeBonheur Comprehensive Epilepsy Program & Neuroscience Institute, LeBonheur Children’s Medical Center, Memphis, TN, USA; 3 Clinical Chief and Director of Pediatric Neurology, St. Jude Children’s Research Hospital, 777 Washington Avenue, P335, Memphis, TN, 38105, USA; 4 Epilepsy Information Service, Comprehensive Epilepsy Program, Wake Forest University, Medical Center Boulevard, Winston-Salem, NC, 27157, USA; 5 Tuberous Sclerosis Alliance, 801 Roeder Road, Suite 750, Silver Spring, MD, 20910, USA; 6 7 Carpenter Close, Ridgefield, CT, 06877, USA; 7 The Comprehensive Epilepsy Management Center, Montefiore Medical Center, 111 East 210th Street, Bronx, NY, 10467, USA; 8 Division of Child Neurology, Department of Neurology, Virginia Commonwealth University School of Medicine, 1001 East Marshall Street, 1st Floor, Richmond, VA, 23298, USA

**Keywords:** West syndrome, Encephalopathic epilepsy, ACTH, Vigabatrin, Infantile spasms, Treatment, Continuum of care, Community resources, Seizures, Infants

## Abstract

**Background:**

Infantile spasms (IS; West syndrome) is a severe form of encephalopathy that typically affects infants younger than 2 years old. Pediatricians, pediatric neurologists, and other pediatric health care providers are all potentially key early contacts for families who have an infant with IS. The objective of this article is to assist pediatric health care providers in the detection of the disease and in the counseling and guidance of families who have an infant with IS.

**Methods:**

Treatment guidelines, consensus reports, and original research studies are reviewed to provide an update regarding the diagnosis and treatment of infants with IS. Web sites were searched for educational and supportive resource content relevant to providers and families of patients with IS.

**Results:**

Early detection of IS and pediatrician referral to a pediatric neurologist for further evaluation and initiation of treatment may improve prognosis. Family education and the establishment of a multidisciplinary continuum of care are important components of care for the majority of patients with IS. The focus of the continuum of care varies across diagnosis, initiation of treatment, and short- and long-term needs. Several on-line educational and supportive resources for families and caregivers of patients with IS were identified.

**Conclusions:**

Given the possibility of poor developmental outcomes in IS, including the emergence of other seizure disorders and cognitive and developmental problems, early recognition, referral, and treatment of IS are important for optimal patient outcomes. Dissemination of and access to educational and supportive resources for families and caregivers across the lifespan of the child with IS is an urgent need. Pediatric health care providers are well positioned to address these needs.

## Background

Infantile spasms (IS; West syndrome) is a severe form of epilepsy of early infancy [1]. Although IS was first described over 160 years ago [1], its diagnosis, evaluation, and management continue to pose many challenges to health care professionals and affected families. Educating pediatricians and general pediatric health care providers about IS may be especially important as a recent IS working group (ISWG) of pediatric neurologists reviewed the literature and determined that early recognition of IS and prompt treatment are mandatory and may improve developmental and cognitive outcomes in some patients [2]. IS presents with myoclonic-tonic seizures (spasms) that may be characterized by flexor, extensor, or mixed movements, a distinct electroencephalogram (EEG) pattern of hypsarrhythmia, and psychomotor delay/arrest [3]. The incidence of IS ranges from 2 to 3.5/10,000 live births, with onset during the first year of life in 90% of those affected. This translates to approximately 2,000 to 2,500 new cases per year in the United States. The peak age of onset is between 3 and 7 months; onset after 18 months is rare, though onset up to 4 years of age has been reported [4]. Spasms usually cease by 5 years of age, but other seizure types are reported in as many as 60% of children with IS even after cessation of spasms [5]. IS occurs in children from all ethnic groups, and boys are affected slightly more often than girls (ratio of 60:40) [6-8].

The etiologic classification of IS includes the categories of cryptogenic and symptomatic. The cryptogenic form of IS occurs in 10% to 40% of IS patients. In infants with cryptogenic IS, no underlying cause is identified and the children have normal development prior to the onset of IS [9]. Outcomes are usually more favorable among children with cryptogenic IS than symptomatic IS, and early effective treatment in cryptogenic cases is associated with improved prognosis [10-16].

Within symptomatic IS, a defined underlying cause is present, usually with developmental delay at onset of spasms. The percentage of IS cases classified as symptomatic has increased over time due to improved diagnostic techniques, such as metabolic and genetic testing and neuroimaging. It is estimated that approximately 60% (in living infants) to 90% (in autopsy neuropathological analysis) of children with IS have an associated underlying disorder that is evident [6,17]. Causes of IS may be prenatal, perinatal, or postnatal. Approximately 50% of cases have a prenatal cause, including central nervous system malformations, intrauterine insults, neurocutaneous syndromes such as tuberous sclerosis complex (TSC), metabolic disorders, or genetic syndromes such as Down syndrome. Identification of genetic associations of IS, a rapidly developing area, suggests IS is a genetically heterogeneous condition involving abnormalities in key developmental pathways in the ventral forebrain and synaptic functional pathways [18]. Perinatal causes include neonatal (hypoxic-ischemic) encephalopathy, and postnatal causes include trauma, infection, and, rarely, tumors. TSC is an important cause of symptomatic IS [19], and the development of IS in children with TSC is closely associated with the development of autistic spectrum disorder in later years [20,21]. The appearance of IS in TSC may be atypical with either the appearance of spasms but no hypsarrhythmia, or the appearance of an abnormal EEG but no spasms. To optimize treatment outcomes, it is recommended that treatment of IS in children with TSC not be delayed while waiting for either hypsarrhythmia to appear or for spasms to begin [22,23].

## Methods

PubMed was searched for IS treatment guidelines, consensus reports, and original research studies. Relevant studies were reviewed to provide an update regarding the diagnosis and treatment of infants with IS. Web sites were searched for educational and supportive resource content relevant to providers and families of patients with IS.

## Results

### Pathophysiology

Little is known about the pathophysiology of IS. The causes of IS appear to be extremely variable [24], and a common mechanism by which all of the different etiologies of IS might converge to lead to spasms has been proposed [25]. Current IS animal models either focus on a specific cause of IS, such as the loss of interneurons (i.e., the *ARX* mouse model), or propose a final common pathway underlying all causes of IS [26]. The stress/corticotropin-releasing hormone (CRH) hypothesis proposes that the common mechanism in all the etiologies of IS causes an increase in the release of stress-activated mediators in the brain, especially the neuropeptide CRH in the limbic and brain stem regions in children with IS [27]. Adrenocorticotropic hormone (ACTH) suppresses the synthesis of CRH, which might explain the treatment efficacy of this stress hormone in IS [27,28]. Other animal models of IS focus on the proposed common pathway of loss of inhibition. The suggested mechanism for the effectiveness of vigabatrin within IS is through its effects as an irreversible inhibitor of γ-aminobutyric acid transaminase (GABA-T) [29].

### Diagnosis

In the great majority of cases, parental observation of spasms initiates the clinical evaluation of IS [30-32]. Parents typically bring the child to the pediatrician for episodes that look like colic or may be mistaken for gastroesophageal reflux. Parental/caregiver videos of infant spasms may assist with the clinical evaluation. Consultation with a pediatric neurologist is warranted as early as possible if the events on video are suspicious. The potential benefits of early diagnosis and treatment cannot be overemphasized as improved neurodevelopment may result [33].

Spasms vary greatly depending upon the muscle groups involved, the intensity of the contraction, and position of the infant during the attack, that is, whether supine or sitting. Spasms may be subtle, brief, and sudden, the most subtle being a head nod or tonic eye rolling, which may be easily missed; they also show great variability in frequency [32,34]. Typically the spasms involve brief symmetrical contractions of musculature of the neck, trunk and extremities lasting up to 5 seconds and occurring in clusters [7,9]. In most cases there is an initial phasic component lasting less than 1 to 2 seconds, followed by a less intense but generally more sustained tonic contraction, which could last up to about 10 seconds. However, in some children this tonic phase may be absent and only the initial phasic component is seen. The number of spasms can vary from a few to more than a hundred per cluster; the duration of a cluster may vary from less than a minute to more than 10 minutes. Among many available web-based videos of IS spasms, the Tuberous Sclerosis Alliance provides an IS informational video depicting spasms, currently found on YouTube at http://www.youtube.com/watch?v=35wRjuvg9MI (see Table 1).

**Table 1:** Organizations providing IS information and family resources

**Organization**	**Website**	**Key resources**
American Epilepsy Society^a^	http://www.aesnet.org	· Provides links to drug assistance programs, treatment guidelines, research publications
Epilepsy Foundation	http://www.epilepsyfoundation.org	· IS fact sheets and awareness
Epilepsy Information Service of Wake Forest Health Sciences^a^	http://www.wakehealth.edu/Neurosciences/Comprehensive-Epilepsy-Center/Epilepsy-Resources.htm	· Epilepsy information toll free hotline, vigabatrin access assistance, support groups
Epilepsy Therapy Project	http://www.epilepsy.com	· IS fact sheets
HopefulCircle.org	http://www.hopefulcircle.org	· A rare diseases online community where patients, caregivers, healthcare professionals, and organizations can come for support and resources
Infantile Spasms Awareness	http://infantilespasmsinfo.org	· IS education and information for pediatricians and parents
Lundbeck, Inc	http://www.lundbeckshare.com	· Comprehensive vigabatrin resource for healthcare providers and patients/families; distribution access to vigabatrin
National Institute of Neurological Disorders and Stroke (NINDS)	http://www.infantilespasms.org	· Infantile spasms information page with overview on IS
National Organization for Rare Disorders (NORD)	http://www.rarediseases.org	· Patient Assistance Programs
		· Online support community, ACTH access assistance
Questcor Pharmaceuticals	http://www.questcor.com	· The Acthar Support & Access Program (ASAP) for prescriptions and reimbursement support related to ACTH treatment
Tuberous Sclerosis Alliance^a^	http://www.tsalliance.org	· Fact sheets on IS in children with TSC
		· IS video (http://www.youtube.com/watch?v=35wRjuvg9MI)
		· Online discussion groups
		· Links to http://www.seizuretracker.com

^a^Organizations that provide resources specifically for health care providers.

In addition to clinical spasms, the defining features of IS include hypsarrhythmia (a specific EEG pattern) and developmental regression. Even a brief EEG recording may confirm the diagnosis, but if IS is suspected, a prolonged awake and asleep video-EEG study is recommended [34]. Interictal (between spasms) EEGs of IS are characterized by hypsarrhythmia as well as chaotic, nonrhythmic, asynchronous, disorganized, high-voltage spike activity and slow-wave activity [9,34]. The hypsarrhythmic pattern is most frequent during stages 2/3 of non-rapid eye movement (non-REM) sleep, followed by waking and arousal, and it does not occur or is greatly reduced during REM sleep [35,36].

The recommended approach to EEG evaluation, during the diagnostic evaluation and during follow-up to determine treatment effectiveness, is an overnight inpatient 24-hour video EEG to capture both hypsarrhythmia and spasms. It will allow the exclusion of other movements that may mimic IS and allow the investigation of other seizure types that may be occurring. If hypsarrhythmia or spasms do not occur, and the events continue at home, the EEG should be repeated in 1 week or as clinically indicated. If developmental regression is present, the EEG should be repeated earlier than 1 week. If an inpatient video EEG is not available, a prolonged 4-hour to 8-hour EEG video during a waking and sleep period may be completed as an outpatient; it is particularly important to capture non-REM sleep.

Once spasms and hypsarrhythmic EEG have been documented, determining the cause of IS becomes the focus of the clinical evaluation [30-32]. The goal is to identify potentially treatable disorders while remembering that early treatment is thought to have an improved developmental outcome in many infants. There are several etiological diagnoses that may respond to specific therapies and lead to resolution of IS (see Table 2). Approximately 30% of children with IS will have no identifiable cause following completion of the history, physical, neurological and ophthalmological (possibly revealing infections, phacomas, and malformations) examination, EEG, and magnetic resonance imaging. Of these remaining 30% of children, a metabolic or genetic etiology will likely be established for fewer than 50%. For these 30% of infants with IS, pyridoxine dosed at 100 mg IV may be administered to screen for pyridoxine-dependent seizures [37]. Pyridoxine should be given during an EEG or an EEG should be repeated following administration of pyridoxine. Additional metabolic evaluation, depending on the individual circumstances, may include urine for organic acids, serum for amino acids, biotinidase determination, lumbar puncture to include neurotransmitters, lactic acid, amino acids, folate metabolites, cerebrospinal fluid glucose, glycine, cells, proteins, IgG index, viral antibody index, and chromosomal studies. Immunoglobulin production can be active for a long time, even years after a primary infection, and infections may represent 10% of IS etiology [38]. A small number of children with IS due to malformations of cortical development, typically involving the posterior quadrant of the brain, are a special subset who may benefit from epilepsy surgery. The remaining children without an identifiable cause will be labelled as cryptogenic.

**Table 2:** Metabolic and other etiological diagnoses that respond to specific therapy

**Diagnosis**	**Specific therapy**
Pyridoxine-dependent seizures	Pyridoxine
Phenylketonuria	Diet
Maple syrup urine disease	Diet
Biotinidase deficiency	Biotin
Menkes disease	Copper histidinate
Hyperammonemia disorders	Possibly diet, depending on which disorder
Nonketotic hyperglycinuria	Benzoate
Tumor	Surgery
Arterial-venous malformation	Surgery
Sturge-Weber syndrome	Surgery if medications fail
Tuberous sclerosis complex	Vigabatrin, ACTH (if vigabatrin fails), and possibly surgery if medications fail
Cortical dysplasias: focal cortical dysplasias, hemimegalencephaly	Possible cortical resection if medications fail
Malformations of cortical development	Epilepsy surgery

Courtesy of Dr Shields.

### Treatment

Recently, an IS consensus group reviewed the most recent practice guidelines from the American Academy of Neurology and the Child Neurology Society for the medical treatment of IS [37], and outlined goals for improving outcomes in IS [2]. The IS consensus group goals for improving IS outcomes include early detection and diagnosis of IS, short-duration treatment with first-line therapy (agreed upon as either ACTH or vigabatrin), timely EEG evaluation of treatment effectiveness, and, if indicated, prompt treatment modification [2]. Early detection of IS is critical. Studies suggest the need for early detection and prompt effective treatment to improve neurodevelopmental outcomes, particularly in cryptogenic cases [10-16]. Evaluation of treatment effectiveness for IS includes cessation of spasms and normalization of the EEG in cryptogenic cases and a resolution of hypsarrhythmia on the EEG in symptomatic cases [34,37,39]. Successful cessation of spasms and resolution of hypsarrhythmia is considered an “all-or-none” response rather than a graded response to treatment [32,39].

The most recent practice guidelines from the American Academy of Neurology and the Child Neurology Society for the medical treatment of IS, which reviewed the available evidence as of 2004, state that ACTH is probably effective and vigabatrin is possibly effective in the cessation of spasms and abolition of hypsarrhythmia [37]. The practice guidelines also state that vigabatrin is possibly effective for children with TSC and IS. Vigabatrin was approved for treatment of IS in the United States in August 2009, and an ACTH gel was approved for treatment of IS in the United States in October 2010. When the practice guidelines were published, there was insufficient evidence to recommend oral corticosteroids or valproic acid as first-line treatments in IS; however, since then, high-dose oral prednisolone has been reported to possibly be effective [12,40].

Children with IS who do not respond to first-line treatments may be considered for epilepsy surgery (only those children with surgical lesions are candidates) or the ketogenic diet, though no controlled trials are available for the efficacy of the ketogenic diet in IS. There currently is insufficient evidence to recommend protocols using new or emerging therapies for IS [37]. The evolution over time of spasms to other forms of epilepsy may then require the use of conventional antiepileptic drugs; however, evidence does not support the clinical efficacy of benzodiazepines, phenobarbital, or most other conventional antiepileptic drugs as effective treatments for IS [37].

#### ACTH

There was consensus among the ISWG that use of ACTH is effective as first-line therapy for IS. Within the United States (US), natural ACTH is used, whereas outside the US tetracosactide, a synthetic ACTH compound, is frequently used. There was insufficient evidence to precisely define the optimum ACTH dose and duration of treatment for IS, although short duration was preferable (i.e., approximately 2 weeks followed by taper) [2]. ACTH is given using intramuscular injection. Effective short-duration treatment may avoid major side effects associated with IS treatment [41]. The most frequent adverse effects associated with short-duration ACTH treatment are irritability, increased appetite leading to weight gain [39], and Cushingoid features. Less frequently seen, but more severe, are hypokalemia and hypertension. Possible serious adverse events include fulminant infections secondary to immunosuppression, glucosuria, and metabolic abnormalities [42]. ACTH followed by long-term treatment with high-dose glucocorticoids in IS patients has been associated with reduced bone mineral density later in life, and such patients may benefit from a calcium-rich diet, monitored vitamin D level, and weight-bearing physical exercise [43]. In all cases, safety measures should be in place (see Table 3). Due to possible immunosuppression, any fever (temperature greater than 101°F rectally) or intercurrent illness should prompt urgent and immediate evaluation by medical personnel. Additionally, live vaccinations should be avoided for 6 months following cessation of therapy due to possible immunosuppression. Similar to high doses of prednisolone, ACTH may suppress the hypothalamic-pituitary-adrenal (HPA) axis resulting in adrenal hypofunction and low cortisol levels. As a result, HPA function should be monitored and hydrocortisone may be needed in patients experiencing stressful situations.

**Table 3:** Suggested safety measures during IS treatment with ACTH and vigabatrin

	**ACTH**	**Vigabatrin**
Baseline hematology	X	X
Baseline serum chemistries (i.e., SMA 20)	X	X
Day 3 serum chemistries (especially potassium)	X	
Twice weekly:		
Blood pressure	X	
Stool guaiac	X	
Urine glucose	X	
Periodic ophthalmic evaluations and visual history		X
Periodic clinical exam and review of potential side effects	X	X

#### Vigabatrin potential adverse events

There was consensus among the ISWG for the use of vigabatrin as effective first-line therapy for IS, particularly in patients with IS and TSC [2]. The consensus stated that the vigabatrin dose should begin at 50 mg/kg/day and be escalated up to 100-150 mg/kg/day in those patients requiring escalation. Efficacy should be assessed within 2 weeks following dose titration. The ISWG consensus report and a review of limited data available from well-controlled clinical trials both state that infants who respond well to therapy with vigabatrin may be continued on the drug for up to 6-9 months with continued ophthalmic evaluation and periodic reevaluation of risk and benefit [2,44]. Vigabatrin is taken by mouth. Among possible adverse events associated with vigabatrin treatment (50-150 mg/kg/day), the most significant is concentric peripheral visual field defects (i.e., pVFD, retinopathy involving loss of peripheral vision in both eyes) [37]. These visual field defects, once present, are permanent and persist even when vigabatrin is discontinued. A recent study of children with IS treated with vigabatrin in early infancy found that 1 in 16 (6%) showed vigabatrin-attributed visual field loss when evaluated at age 6 to 12 years [45]. The study [45] needs to be replicated in a larger number of children treated in infancy and old enough to cooperate with detailed testing to confirm these preliminary data. The duration of therapy, cumulative dose, and daily dose have been implicated as risk factors for visual field changes with vigabatrin use [46]. Accurate assessment of visual field changes in infants is challenging. A recent expert consensus protocol for visual evaluation for infants on vigabatrin presents recommendations for visual function evaluations by the child’s developmental age and/or ability, to be performed by neurologists and ophthalmologists [46]. A history of the patient’s visually-oriented behavior (e.g., bumping into objects or ignoring objects in the environment) should be obtained from parents or caregivers [46,47]. Because infants and young children with IS are unable to perform perimetry, confrontation testing in which small toys of interest are held in the peripheral field to see if any eye movement occurs is recommended as a qualitative assessment to identify patients who may have pVFD and may require additional testing [46,47]. A full-field electroretinogram (ERG) is recommended as the primary screening modality for infants and children younger than 2 years, however, the risk of required sedation needs to be considered for each patient [46]. A visual history should be performed at every clinic visit in children treated with vigabatrin. In the US, vigabatrin is available only under a special restricted distribution program (i.e., the Support, Help and Resources for Epilepsy [SHARE] program; http://www.lundbeckshare.com). Children on vigabatrin are required to have periodic ophthalmic evaluations beginning with a baseline evaluation at initiation of therapy (no later than 4 weeks after starting treatment) and at least every 3 months while on therapy, as well as 3 to 6 months after cessation of treatment (see Table 3). Other adverse side effects with vigabatrin therapy include sedation, irritability, insomnia, and hypotonia [48-51].

## Discussion

### Establishing a continuum of care

#### Diagnosis

Education about IS, treatment options, and a continuum of care is an ongoing process begun at the first contact with the child and family during the clinical evaluation and diagnosis, and continued as the treatment plan is developed. The methods for establishing a continuum of care with close follow-up will vary depending on medical center and physician preferences, and comorbid conditions of the infant with IS. In addition to general information about IS etiology, prognosis, and any issues associated with comorbid disorders, parents/caregivers should be educated about treatment options in order to make informed decisions. Detailed information regarding administration of medication and possible side effects, as well as who to contact if there are problems or questions, and when to make contact, should be discussed. Parents/caregivers would benefit from information regarding resources that assist with access to treatment (see Table 1). The Acthar Support and Access Program (A.S.A.P.) and the National Organization for Rare Disorders (NORD) may be especially helpful for access to Acthar Gel. The SHARE program, developed to assist with access to vigabatrin in the US, provides information about vigabatrin and provides prescription support. In the US, vigabatrin is currently only available through the SHARE Program due to the potential adverse risk of permanent vision loss.

#### Initiation of treatment

Parental/caregiver education and training is required to prepare for administering treatment at home [52]. Before initiating treatment at home, parents/caregivers should have all relevant emergency contact phone numbers and the plan of contact should be determined. For example, it should be clarified whether the treating pediatric neurologist is the direct contact for parents/caregivers or the general pediatrician is the first contact and will consult with the pediatric neurologist as needed. The availability of a home health nurse in the early transition from the hospital to home care can be very helpful for parents/caregivers to ensure adequate training in treatment administration, to relieve stress, and to provide support.

#### Short-term needs

Following the transition from treatment in the hospital to home care, the health care provider network for the child with IS and the overall medical and psychosocial treatment plan should be discussed. All health care providers who will be involved in the child’s care should be identified and the specific treatment plan should be determined. Discussion of potentially useful strategies to manage side effects such as irritability will help alleviate caregiver stress and may improve treatment adherence. Especially important is the identification of specific adverse effects that caregivers should look for that indicate the need to immediately contact the treating physician.

Once the treatment plan has been established, discussion of multidisciplinary resources for children with IS and their families is needed due to potentially lifelong needs related to medical care and poor developmental outcomes. Parents and caregivers need to be aware of available coping resources to assist with the emotional and psychosocial impact of the IS diagnosis (see below). This is particularly important as parent/caregiver distress may interfere with the ability to comply with providing medical care to the child at home.

#### Long-term needs

Given the possibility of poor developmental outcomes in IS, including the emergence of other seizure disorders and cognitive and developmental problems [7,53], the establishment of a long-term multidisciplinary continuum of care for children with IS is important. With the possible exception of children diagnosed with cryptogenic IS who show cessation of spasms and hypsarrhythmia in response to treatment, access to and evaluation by a variety of professionals, such as child neurologists, developmental pediatricians, child psychiatrists, pediatric nurse practitioners, nurses, specialists in rehabilitation services (physical, occupational, and speech therapy), vocational rehabilitation counselors, neuropsychologists, social workers, pharmacists, and others is needed. Long-term neuropsychological and psychological evaluation may be especially critical for assessing developing cognitive and psychosocial abilities important for the patient’s function and to identify areas in need of intervention or external support. The comprehensive ‘village’ of resources should include the integration of resources from health care practitioners, social service professionals, and community agencies [54].

Children with IS often require an evaluation for early intervention programs for developmental impairment. Because available resources vary by community and by state, parents/caregivers should ask the treating physician about local services. Additionally, parents/caregivers can search for services using the Internet, ask about listings of social services at their local library, and determine whether their county of residence publishes information about social services and resources relevant for IS and comorbid disorders. Parents/caregivers are encouraged to create a support network and engage family and friends in the search for information and services. Persistence is important; if initial contacts do not lead to desired services, parents/caregivers should seek assistance from alternative service organizations. The optimal management of IS is complex, and care across the lifespan may be complicated by ongoing seizures, intellectual disability and learning disorders, or behavioral and/or psychological issues. Adults who have a history of IS may require discussion of future guardian issues, group home applications, and respite care options [54].

### Resources for families and health care providers

#### IS overview and support resources

Table 1 lists organizations that provide information about IS and provide family and health care provider resources. Given the relative rarity of IS, these online resources allow widespread access to IS medical information by physicians and families and provide web-based support forums for families. Available resources include fact sheets about IS, including information about the disorder, treatment options and outcomes, and online communities; forums for parents/caregivers of children with IS to communicate with one another and provide support. Family caregivers should be cautioned that medical information obtained within an online community should be discussed with the treating physician to verify its accuracy.

#### Treatment and health care provider resources

Assistance programs for children with IS help uninsured and under-insured families gain access to medications and financial support (premium/co-payment assistance program) by working with pharmaceutical companies and insurance plans (see Table 1). Assistance with physician referrals is also available. For example, the Tuberous Sclerosis Alliance has created a provider list for TSC clinics. Health care provider resources are also available, including links to treatment guidelines, research publications, and online community discussion groups for medical professionals.

## Conclusions

IS imposes a significant ongoing challenge to the child’s family and caregivers, as well as to health care professionals. Early detection and referral to a pediatric neurologist for clinical evaluation and prompt effective treatment is strongly recommended as it may improve prognosis. To best navigate the medical environment and optimize clinical care, the child’s family and caregivers need access to up-to-date information about IS, effective treatments, and establishment of a multidisciplinary continuum of care, which includes access to resources for psychosocial support. Further dissemination of and access to educational and supportive resources for families and caregivers across the lifespan of the child with IS is an urgent need.

## Competing interests

The development of this manuscript was supported by an unrestricted grant from Questcor Pharmaceuticals, Union City, CA. The supporter was not in any way involved in the development of the scientific content of this manuscript.

Dr Wheless has received grants from NIH, the Shainberg Foundation, UCB, Ovation, Questcor, Marinus, Ortho-McNeil, King, Cyberonics, and Eisai; is a consultant for UCB, Ovation, Questcor, Marinus, Ortho-McNeil, King, Cyberonics, Pfizer, Eisai, Valeant, CyDex, and Neurelis; is a member of a speakers bureau for UCB, GlaxoSmithKline, Ortho-McNeil, Cyberonics, Pfizer, Eisai, Shire, and Valeant; and has received honoraria and reimbursement for travel expenses from Questcor.

Ms Gibson has received grants from Questcor, Ovation, Valeant, Eisai, Ortho-McNeil, and GlaxoSmithKline; and has received honoraria and reimbursement for travel expenses from UCB, Questcor, Ovation, and Sepracor.

Ms Rosbeck has received reimbursement for travel expenses from Novartis Oncology. The Tuberous Sclerosis Alliance has received grants from Lundbeck, Questcor, Novartis Oncology, UCB, and Cyberonics.

Ms Hardin, now retired, was formerly Vice President of Patient Services at the National Organization for Rare Disorders, where she had oversight of the Acthar Gel Patient Assistance Program and the Infantile Spasms Co-payment Assistance Fund, both of which received support from Questcor Pharmaceuticals.

Ms O’Dell is a consultant for and has received honoraria and payment for development of education presentations from Ovation and Questcor; and has received grants and reimbursement for travel expenses from Questcor.

Dr Whittemore has received grant funding from NIH, and has received reimbursement for travel expenses from Novartis Oncology. The Tuberous Sclerosis Alliance has received grants from Lundbeck, Questcor, Novartis Oncology, UCB, and Cyberonics.

Dr Pellock is a consultant for Eisai, Jazz, King, KV, Marinus, NeuroPace, Ortho-McNeil/Johnson & Johnson, Lundbeck, Pfizer, Questcor, UCB, and Valeant; has participated in an advisory board for Eisai, Ortho-McNeil/Johnson & Johnson, Lundbeck, Questcor, UCB, and Valeant; is a lecturer for Eisai, Ortho-McNeil/Johnson & Johnson, Lundbeck, Questcor, UCB, and Valeant; is a researcher for Eisai, Marinus, Ortho-McNeil/Johnson & Johnson, Lundbeck, Pfizer, Questcor, UCB, and Valeant; and has received honoraria and reimbursement for travel expenses from Questcor.

## Authors’ contributions

JWW contributed to the conception and design of the paper, contributed to the drafting of the paper, contributed to revising the paper, and gave approval of the final version. PAG contributed to the conception and design of the paper, contributed to the drafting of the paper, contributed to revising the paper, and gave approval of the final version. KLR contributed to the conception and design of the paper, contributed to revising the paper, and gave approval of the final version. MH contributed to the conception and design of the paper, contributed to revising the paper, and gave approval of the final version. CO contributed to the conception and design of the paper, contributed to the drafting of the paper, contributed to revising the paper, and gave approval of the final version. VW contributed to the conception and design of the paper, contributed to revising the paper, and gave approval of the final version. JMP contributed to the conception and design of the paper, contributed to revising the paper, and gave approval of the final version.

## Pre-publication history

The pre-publication history for this paper can be accessed here:

http://www.biomedcentral.com/1471-2431/12/108/prepub
